# Targeting of Extracellular Vesicle-Based Therapeutics to the Brain

**DOI:** 10.3390/cells14070548

**Published:** 2025-04-04

**Authors:** Anastasia Williams, Heather Branscome, Fatah Kashanchi, Elena V. Batrakova

**Affiliations:** 1 Laboratory of Molecular Virology, School of Systems Biology, George Mason University, Discovery Hall Room 248, 10900 University Blvd, Manassas, VA 20110, USA; awill57@gmu.edu (A.W.); hbransco@gmu.edu (H.B.); fkashanc@gmu.edu (F.K.); 2 American Type Culture Collection (ATCC), Manassas, VA 20110, USA

**Keywords:** extracellular vesicles, drug delivery, brain

## Abstract

Extracellular vesicles (EVs) have been explored as promising vehicles for drug delivery. One of the most valuable features of EVs is their ability to cross physiological barriers, particularly the blood–brain barrier (BBB). This significantly enhances the development of EV-based drug delivery systems for the treatment of CNS disorders. The present review focuses on the factors and techniques that contribute to the successful delivery of EV-based therapeutics to the brain. Here, we discuss the major methods of brain targeting which includes the utilization of different administration routes, capitalizing on the biological origins of EVs, and the modification of EVs through the addition of specific ligands on to the surface of EVs. Finally, we discuss the current challenges in large-scale EV production and drug loading while highlighting future perspectives regarding the application of EV-based therapeutics for brain delivery.

## 1. Introduction

There is a dire, urgent, and unmet need for the efficient targeting and delivery of various potent therapeutics to the brain to treat disorders of the central nervous system (CNS). It is estimated that 3.4 billion people worldwide, or 43% of the population, are affected by neurological conditions and, furthermore, that these conditions were responsible for approximately 11.1 million deaths from 1990 to 2021 [1]. Globally, the number of people living with or dying from neurological conditions has risen substantially over the last 30 years; this is mainly due to the aging population as well as environmental and lifestyle risk factors [2]. Some nervous system conditions cause lifelong disability, whereas others are associated with high fatality rates. Therefore, delivery of therapeutics to the brain at the sites of tissue injury, tumor, or infection with limited toxicity is the imperative goal for successful pharmaceutics.

The delivery of therapeutics to the brain through systemic circulation is hindered by three physiological barriers that isolate the brain from the periphery. One is the blood–brain barrier (BBB), which restricts drug diffusion from blood circulation to brain parenchyma at the level of the brain microvessel endothelium (BME) [3,4]. The tight extracellular junctions of the BME cells, along with relatively low pinocytic activity, enzymatic barriers, and the expression of various efflux protein systems restrict access to the brain for most substances circulating in the blood. The second is the blood–cerebrospinal fluid barrier (BCSFB) which prevents drug diffusion from blood to the cerebrospinal fluid (CSF) at the choroid plexus and meninges [5,6]. The third barrier that drugs encounter is the ependyma which isolates the brain parenchyma from the CSF [7]. Finally, one additional barrier that remains a major limitation for the delivery of potent antineoplastic therapeutics is the blood–brain tumor barrier. In this regard, in addition to the BBB, the blood–brain tumor barrier represents a challenging obstacle for effective drug delivery. For example, glioblastoma multiforme (GBM) is the most aggressive malignant tumor affecting the brain with a five-year survival rate of about 5%, as most chemotherapeutic treatments are not able to pass the protective CNS barriers [8]. Thus, tumors of the CNS, in particular GBM, remain largely incurable [9].

Successful treatment of many debilitating CNS disorders requires efficient drug delivery systems that can overcome the physiological barriers described above. These disorders include stroke [10,11], neurological diseases such as Alzheimer’s and Parkinson’s [12,13,14], infectious diseases (e.g., meningitis, encephalitis, prion disease, and HIV-related dementia) [15,16,17,18,19,20], and brain tumors [21,22]. Other disorders, such as depression [23], chronic migraine [24], fungal infections [25], obesity [26], and lysosomal storage diseases [27] require drug transport to the brain as well. More recently, newly emergent conditions, such as cognitive impairment following SARS-CoV-2 infection, have become an additional burden worldwide [28]. For these reasons, the efficient treatment of CNS disorders remains a challenging task.

Extracellular vesicles (EVs), including exosomes and microvesicles, have emerged as promising tools for the treatment and diagnosis of neurodegenerative diseases. This interest stems from their role in intercellular communication and their ability to overcome natural physiological barriers, including the BBB. In recent years, innovations in analytical imaging techniques have expanded research on EVs through increased understanding of the chemical function and structure of EVs. These innovations have provided insight into the biological roles of EVs such as compartmentalization, storage, and molecular trafficking [29]. EVs play a crucial role in cell-to-cell communication through the transfer of proteins, lipids, and nucleic acids, which collectively regulate a variety of physiological and pathological processes. These naturally equipped nanocarriers hold great promise as a safe and biocompatible drug delivery system with low immunogenicity, high stability in circulation, and the ability to cross physiological barriers [30,31,32]. Additionally, EVs can be engineered to express a targeting moiety on their surface or encapsulated within their membranes to further supplement their biological activity. Thus, EVs offer the benefits of synthetic nanocarriers and cell-mediated drug delivery systems while at the same time overcoming many of their limitations.

**Figure 1:**
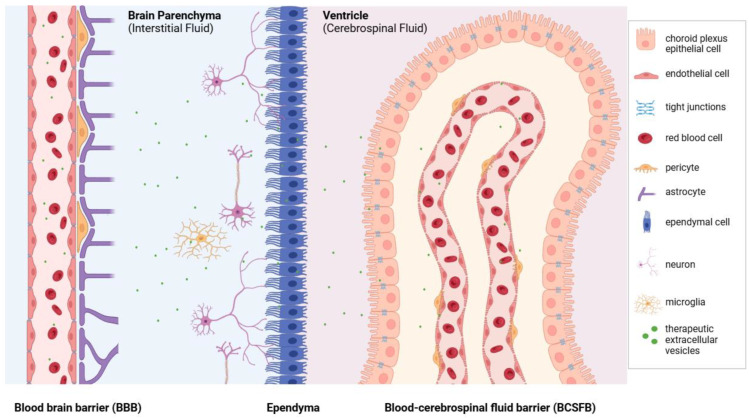
Barriers of drug delivery to the brain. The brain is protected by three physiological barriers: the blood–brain barrier (BBB), the ependyma, and the blood–cerebrospinal fluid barrier (BCSFB). These barriers effectively prevent many therapeutic agents from reaching the brain, complicating treatment efforts for central nervous system (CNS) disorders. However, extracellular vesicles (EVs) have demonstrated the ability to bypass all three of these barriers, making them promising candidates for use as delivery systems for CNS therapeutics.

Regarding the CNS, EVs have been shown to cross all three barriers of the brain (i.e., BBB, BCSFB, and ependyma), thus highlighting their therapeutic potential in neurological conditions (Figure 1). Although the mechanisms of EV transport across these barriers are still not fully understood, the current literature provides insights into their movement and function. For example, there is mounting evidence of the bidirectional transportation of EVs through the BBB, in which EVs are transmigrated through the endothelial cells via endocytic pathways and adsorptive and receptor-mediated transcytosis [33,34,35,36]. These processes allow EVs to effectively move between the bloodstream and the brain parenchyma. Similarly, EVs have been shown to transmigrate across the BCSFB through transcytosis [37,38,39,40], enabling their movement between blood vessels and CSF. Once in the CSF, EVs can cross the ependyma into the brain parenchyma [40,41]. While the mechanism of EV transport across the ependyma is ill defined, there is evidence of selective transportation; for instance, Grapp et al. demonstrated that folate receptor α (FRα)-positive EVs crossed the ependyma preferentially to FRα-negative EVs [40].

Although the native characteristics of EVs make them ideal for drug delivery, their therapeutic implementation remains challenging due to their heterogenicity, lack of standardized isolation and purification methods, complex characterization, and unknown therapeutic mechanisms. This is further compounded by underdeveloped large-scale production protocols and insufficient clinical grade production [42]. Other limitations include issues related to biogenesis, composition, dosing, and the formulation of EVs. As such, more thorough investigations and utilization of advanced production and analytical techniques are needed to improve EV manufacturing strategies. Furthermore, efficient drug loading without disrupting the EV surface architecture, which plays a crucial role in the delivery of their cargo to target cells, remains challenging [42]. Regarding the successful treatment of CNS disorders, one major drawback for the therapeutic application of EVs is their inadequate targeting ability to the brain. In most studies, systemic administration of EVs resulted in preferential accumulation in the liver and spleen [43,44,45]. Therefore, strategies that allow accumulation of significant amounts of EV-based therapeutics in the brain are crucial.

This review underscores the importance of EV targeting the CNS for therapeutic purposes. Active targeted drug transport to disease sites offers several advantages, including improved drug efficacy, prolonged half-life, time-controlled drug release, and diminished immunogenicity and cytotoxicity. Here, we have described the various administration routes for EV-based therapeutics and have detailed the factors influencing successful delivery of EV-based therapeutics to the brain. We have also reviewed various studies concerning CNS targeting based on the nature of EV origin, as well as the development of designer EVs with moieties directed to specific brain cells and tissue types. Lastly, we have focused on external effects that guide EV-based formulations to the brain.

## 2. Importance of Administration Routes

**Figure 2:**
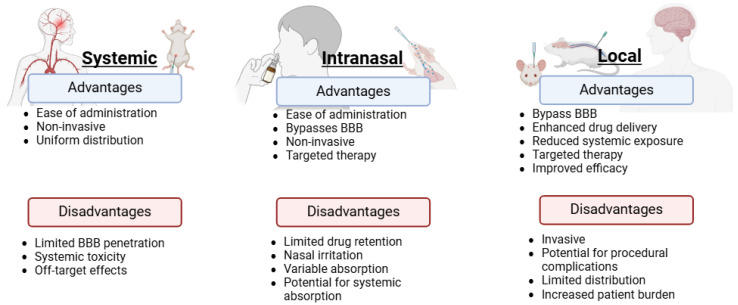
Routes of administration to the CNS. Drug delivery to the CNS remains a challenge due to the complex physiology of the CNS which regulates access to the brain, such as the BBB. The major administration routes for drug delivery to the brain include systemic delivery in which therapeutics traverse through the blood stream and must pass through the BBB, invasive local delivery, and intranasal delivery in which therapeutics bypass the BBB, but efficiency is subject to the nasal mucus layer. This graphic indicates the major advantages (blue) and disadvantages (red) of these three routes of delivery.

With the physiological barriers to the brain, delivery of therapeutics to the CNS remains a challenge. Currently, there are three major routes of EV-based drug delivery: systemic administration, local administration, and intranasal administration. Each route of administration poises unique challenges and advantages (Figure 2).

### 2.1. Systemic Administration

Systemic administration of CNS-targeted therapeutics requires effective transport across the BBB, which significantly restricts penetration of therapeutic agents to the brain from the periphery. The BBB is universally considered the main barrier for drug delivery due to its large blood–brain interface, with an estimated 98% of potent molecules deemed clinically ineffective due to their inability to cross the BBB [46]. Emerging data have shown that EV-based formulations have unique features which permit the crossing of biological barriers and transfer of therapeutic cargo to the disease site. Thus, a number of studies have reported successful systemic delivery of EV-based therapeutics to the brain, which resulted in significant therapeutic effects in different models of neurodegenerative disorders [45,47,48,49,50,51,52,53,54,55].

The well-documented literature indicates that systemic treatment with EVs released by multipotent mesenchymal stromal cells (MSCs) induces neurovascular remodeling and significantly improves neurologic outcomes in various models of neurodegeneration. For instance, in a traumatic brain injury (TBI) rat model, intravenous (IV) injection of MSC-generated EVs were shown to promote both neurovascular remodeling and functional recovery [50]. This was further shown with improved spatial learning and sensorimotor functional recovery compared to PBS control. In another study, systemic administration of MSC-EVs reduced inflammation and cell apoptosis, promoted angiogenesis, and improved functional recovery after spinal cord injury (SCI) in rats [51]. Similarly, the systemic administration of MSC-generated EVs was shown to significantly improve functional recovery in stroke rats compared with PBS controls [55]. Adult male Wistar rats with middle cerebral artery occlusion (MCAo)-induced stroke treated with MSC-EVs through tail-vein administration displayed significant improvement in functional recovery compared with PBS-treated controls. It was also demonstrated that the miRNA content (i.e., microRNA 133b (miR-133b)) of the MSC-EVs was responsible for the modulation of neurovascular plasticity and neurological recovery from stroke [52]. Finally, IV-injected allogenic adipose-derived MSC-EVs have also significantly protected the brain against sepsis-induced injury in rats by decreasing the levels of inflammatory mediators TNF-α, IL-1β, NF-κB, and matrix metallopeptidase 9 [56]. Administration of MSC-EVs decreased endothelial cell apoptosis and production of the anti-inflammatory mediators IL-6 and IL-10. Overall, naturally produced MSC-derived EVs, without loaded therapeutic agents, may serve as a cell-free therapeutic by creating a tolerogenic immune response for the treatment of autoimmune and CNS disorders.

Incorporation of potent drugs into EVs could further enhance their therapeutic effects. For example, EV nanocarriers accomplished successful delivery of brain-derived neurotrophic factor (BDNF) following IV injection in mice [48]. Furthermore, Yuan et al. demonstrated that EV delivery to the CNS was improved with neuroinflammation, a typical condition associated with CNS disorders. A similar study demonstrated that intraperitoneal (IP) administered EVs loaded with lysosomal enzyme tripeptidyl peptidase-1 (TPP1) accumulated in the brain of a late-infantile neuronal ceroid lipofuscinosis (LINCL) mouse model [49]. Additionally, LINCL mice who received treatment with these TPP1 EVs were shown to have an increased lifespan compared to the control. Moreover, a single IV dose of MSC-EVs along with copper sulfide nanoparticles (CuSNPs) resulted in the amelioration of the oxidative stress and inflammatory markers in rats with cadmium (Cad)-induced neurological disorder [54]. Finally, significant therapeutic effects of quercetin (Que) loaded into blood-borne EVs were reported in a rat model of Alzheimer’s disease (AD) [57]. Que is a flavonoid natural compound that has been recognized as a promising cognitive enhancer with neuroprotective, anti-oxidative, and anti-inflammatory effects. It was also reported that IV administration of EV-Que resulted in the inhibition of cyclin-dependent kinase 5 (CDK5)-mediated phosphorylation of Tau and a reduction in the formation of insoluble neurofibrillary tangles (NFTs) in the AD rat model.

Regarding the application of EV-based formulations for the treatment of brain tumors, several studies have shown promising outcomes upon their systemic administration. In one study, glioma-bearing rats were injected through an IV route with EV-like nanoparticles isolated from ginseng (*Panax ginseng*, Gen-EVs). These formulations contained various bioactive compounds (i.e., miRNAs, proteins, lipids, ginsenosides) that incited an immune response [58]. Here, Kang and Min showed that ptc-miR396f-mediated targeting of genes related to apoptosis in Gen-EVs imparted significant gene silencing on proto-oncogene c-MYC, which resulted in tumor suppression and increased the survival rate of glioma-bearing animals. Additionally, this study demonstrated that treatment with Gen-EVs reduced pro-tumoral cytokines, promoted induction of T cells, recruited M1 macrophages, and suppressed regulatory T cells (Tregs) within the tumor-microenvironment (TME). Of note, intracranial (IC) injections of Gen-EVs resulted in more efficient and prolonged effects compared to IV injections, demonstrating the importance of route of administration in these studies.

In another model, plant-derived EVs were also used for glioma treatment in Wistar rats with orthotopically implanted C6 glioma cells, in which Gen-EV treatment was associated with a reduction in tumor volume compared to the PBS control [59]. It was hypothesized that Gen-EVs were able to infiltrate tumors through macropinocytosis due to the overexpression of glucose transporter 1 (GLUT-1) in C6 glioma cells. Along these lines, IP administration of miR-124a-loaded EVs derived from bone marrow MSCs led to a 50% increased lifespan of mice with GBM(GSC267), and no evidence of the tumor was found on the histological analysis of surviving animals [53]. It was suggested that miR-124a acts by silencing Forkhead box (FOX)A2, thereby resulting in aberrant intracellular lipid accumulation.

Systemic administration of therapeutics is relatively easy to execute and can be conducted frequently, thereby allowing for a wider range of dosing schedules. However, unmodified systemically administered EVs show preferential accumulation in the organs that are primarily considered as a part of the reticuloendothelial system (RES). These organs contain a large population of phagocytes, which are known to rapidly accumulate and clear drug nano-formulations. Studies have suggested that tissue-resident macrophages have an important role in taking up EVs after systematic injection [60,61]. Thus, a number of reports indicate predominant localization of systemically administered unmodified EVs in the spleen, liver, kidney, gastrointestinal tract (GI), and lungs [45,49,62,63,64]. Specifically, imaging of mice with IV-injected reporter EVs demonstrated that EV distribution went through two phases [62]. The first phase was described as a rapid distribution phase, in which the EVs were found throughout the mice with a peak period at approximately 30 minutes (min) for many tissues (including the brain). The second phase was descried as an elimination phase in which EVs were removed through the hepatic and renal systems, lasting over 360 min (with levels in the brain reaching base line at 120 min) [62].

Even within systemic administration there are differences in EV accumulation. For instance IV-injected EVs were found to accumulate within the liver and spleen in higher amounts than either IP or subcutaneous (SC) injections, which accumulated more in the pancreas [62]. Similarly, the biodistribution of cancer cell-derived EVs (HEK293T cells) administered via three different systemic delivery routes (i.e., IV, IP, SC) was investigated in mice [63]. In contrast to IV injections, IP and SC injections resulted in significantly lower EV accumulation in liver, whereas an increased accumulation was observed in the pancreas and GI tract. Importantly, the accumulation levels of EVs in the brain were negligible compared to the peripheral organs. A similar trend was observed in non-human primates (NHPs), healthy adult rhesus macaques [44]. In other studies, the liver, kidney, and spleen had the highest levels of autologous monocyte-derived EVs administered through IV or IP routes, and the accumulation levels of systemically injected EVs in the brain was lower compared to other organs [48,65,66]. This highlights the need for innovative and clinically viable drug delivery systems using alternative administration routes.

### 2.2. Local Administration

The most straightforward approach to delivering drug formulations to the brain is intracerebral injections. A considerable number of studies have reported efficient brain delivery of EV-based formulations injected orthotopically for the treatment of neurodegenerative disorders. EVs loaded with miR-124 have been engineered to study a mouse model of Huntington’s disease (HD) as miR-124 has been shown to be downregulated in patients with HD [67]. Here, miR-124 formulations were injected into the striatum of R6/2 transgenic HD mice; as a result, the EV-treated mice exhibited higher levels of miR-124 expression compared to the non-treated control animals. Interestingly, the treatment with EV-miR124 did not produce significant behavioral improvements. In a similar study, the development of EV-based formulations with small interfering RNAs (hsiRNAs) targeting Huntingtin mRNA has also been reported [68]. In this study, hsiRNA-loaded EVs were shown to silence up to 35% of Huntingtin mRNA when unilaterally infused into the mouse striatum.

Furthermore, therapeutic effects of MSC-derived EVs were investigated in a mouse model of AD [69]. Injections of MSC-EVs into the hippocampus stimulated neurogenesis in the subventricular zone and alleviated amyloid-β peptide (Aβ1)-induced cognitive impairment. Similar outcomes were also reported for neuroblastoma-derived EVs, in which neuroblastoma-derived EVs were shown to trap Aβ when exogenously injected into the brains of transgenic mice [70]. These Aβ-collecting EVs were then phagocytosed by microglia. Furthermore, this study demonstrated that continuous administration of EVs decreased Aβ levels and amyloid depositions [70]. Therefore, it was suggested that intracerebrally administered EVs acted as potent scavengers for Aβ by carrying it on the EV surface.

Moreover, ipsilateral hippocampus injections were utilized to study the ability of EVs to achieve brain delivery of adeno-associated virus serotypes (AAV6 and AAV9) encoding GFP in mice [71]. Three months following injection, elevated levels of GFP expression of both serotypes were detected within neurons and oligodendrocytes, and only a low expression was observed within astrocytes. Furthermore, the effect of MSC-derived EVs in mice with lipopolysaccharide (LPS)-induced brain inflammation was investigated upon intraventricular administration [72]. MSC-EVs reduced hippocampal reactive astrogliosis and ameliorated inflammation-induced learning and memory impairments in mice. Finally, stem cell-derived EVs reduced neuroinflammation, rescued cortical damage, and improved the motor deficits when EVs were injected into the rat brain in a rat TBI model [73]. The proposed mechanism of the therapeutic effects of stem cell-derived EVs may, in part, relate to their immunomodulatory properties, which are responsible for shifting the polarization of microglia from the M1 to the M2 phenotype.

Local administration was also utilized for the treatment of brain tumors. A local administration of miR-146b that reduces GBM cell motility and invasion was suggested to treat brain tumors. EVs derived from miR-146b-expressing MSCs were shown to attenuate glioma growth in a primary brain tumor rat model when injected intratumorally [74]. Next, brain endothelial cell-derived EVs were shown to deliver anticancer drugs across the BBB for the treatment of brain cancer in a zebra fish model [75]. EVs loaded with doxorubicin (DOX) and paclitaxel were injected into the brain ventricle and EV treatment significantly decreased the fluorescent intensity of Xeno-transplanted cancer cells and tumor growth markers.

Although the administration of EV-based formulations directly into the brain avoids entrapment and clearance by RES, this approach is highly invasive and can cause a variety of side effects, including muscle tremors, brain lesions, and behavioral changes. Additionally, only an exceedingly small volume of a drug solution can be injected directly into the brain. For example, only 1 to 2 μL of treatment solution could be used in mouse models. Therefore, other administration routes may be preferentially considered for avoiding RES and delivering a therapeutically significant amount of drugs to the brain.

### 2.3. Intranasal (IN) Administration

One novel approach to address the challenge of bypassing the BBB is to inject drugs intranasally (IN) [76]. Intranasal administration provides a non-invasive, safe, and efficient path to the brain for EV therapeutics. A significant bulk of the literature has indicated the usefulness of the IN administration route for the brain delivery of EV-based drug formulations [44,45,47,77,78].

Specifically, the successful brain targeting of EV-based formulations of anti-inflammatory agents such as catalase or curcumin via IN administration was reported in several manuscripts [47,77,79]. In one such study, fluorescently labeled EVs were administered intranasally in a mouse model and imaged 30 min after the treatment [79]. EVs were shown to be diffused across the brain, with a high concentration located within the olfactory bulb, suggesting rapid translocation of EVs to the brain. Furthermore, this study suggested that EVs loaded with curcumin accumulated in microglial cells and protected mice from LPS-induced brain inflammation. In another similar study, IN-administered EVs were found throughout the brains of treated mice, with localization in the cerebral frontal cortex, central sulcus, and cerebellum [47]. It was hypothesized that the IN administration provided drug transport directly to the brain from the nasal cavity along the olfactory and trigeminal nerves. In addition, brain delivery of catalase-loaded EVs through the IN route was shown to increase neuronal survival and significantly reduce inflammation in a mouse model of Parkinson’s disease (PD). Remarkably, catalase-loaded EVs, but not empty EVs, were detected throughout all sections of the brain followed by daily administration for four weeks [77]. Finally, EVs containing miR-219 were shown to promote oligodendrocyte precursor cell (OPC) differentiation and improve remyelination in aging rats upon IN administration [78]. Notably, in addition to EVs that were located in the brain, significant amounts of EVs were also found in peripheral organs, including the liver, kidney, and lungs [44]. Overall, the IN route of administration holds promise as a therapeutic approach for addressing neurological disorders.

In conclusion, the delivery method is one of the most critical issues that must be considered in the development of EV-based therapeutics for the CNS. The route of administration should be assessed for clinical use, as the administered route can define the tissue distribution of the treatment EVs and aid in the targeting of diseased tissue. Understanding the distribution of EVs associated with an administration route can help optimize therapeutic dose, treatment regimes, and inform regulations for clinical applications.

## 3. Capitalizing on the Biological Origin of EVs

### 3.1. Organotropism of EVs

The organotropism of EVs plays a pivotal role in organ distribution [47,80,81]. It has been shown that EVs preferentially fuse with the same cell type as their parental cells rather than other types of resident cells in the receptor organ. It was hypothesized that various integrins expressed on the surface of EVs may determine their organotropism [82]. This feature could be used for targeted delivery of potent therapeutics to the brain for treatment of various neurodegenerative disorders, with collected work summarized in Table 1.

In this regard, neuronal cell-derived EVs may provide improved drug delivery to the CNS. For instance, EVs derived from human neural stem cells (hNSCs) have been examined in different stroke models [83,84,85]. One such study demonstrated functional improvement in a pig ischemic stroke model, in which hNSC-EV treatment promoted neuronal tissue preservation [83]; another study showed improved motor function in a murine stroke model [84]. To further enhance the therapeutic capabilities of hNSC-EVs seen in these studies, Zhang et al. stimulated donor hNSCs with the proinflammatory factor interferon gamma (IFN-γ) [85]. In this study, hNSC-EVs were injected into the ischemic regions of ischemic stroke rats at 24 h (hrs) after stroke onset. Both the hNSC-EV and IFN-γ-hNSC-EV-treated groups showed significant improvement in neurological functional recovery compared to those in the PBS-treated control group. However, the functional outcomes in the IFN-γ-hNSCEV-treated group were superior to those in the hNSC-EV-treated group.

According to another report, hNSC-EVs were also shown to alleviate chronic neuroinflammation and cognitive impairments induced by peripheral LPS challenge in mice upon IN administration [86]. The hNSC-EV-treated mice displayed reduced activation of microglia, NLRP3 inflammasomes, proinflammatory cytokines, and the levels of neurogenesis were similar to those of age-matched controls. Importantly, IN-administered hNSC-EVs were detected in neurons and microglia in various brain regions in LPS-treated mice. Next, the ability of neuronal EVs to capture and clear amyloid-β peptide (Aβ) aggregates that contribute to AD pathogenesis was investigated, in which EVs derived from neuronal and glial cells were assessed [87]. Here, EVs were administered through intracerebral infusion into the brains of AAPP transgenic mice. Neuronal EVs alone were shown to decrease Aβ and amyloid depositions. Notably, neuronal EVs, but not glial EVs, had abundant glycosphingolipids, which are essential for Aβ binding and assembly, on the EVs. The impact of EV organotrophic features on the treatment of stroke was also investigated in a murine thromboembolic (TE) stroke model [84]. In this report, therapeutic effects of EVs released by neural stem cells (NSCs) and MSCs were compared, in which mice were treated with three doses of EVs or PBS through tail-vein injection. NSC-EVs were more effective in improving cellular, tissue, and functional outcomes (such as improved motor function) compared to MSC-EVs, although both NSC-EVs and MSC-EVs were similar in protein markers and structure. Thus, clear benefits of NSC-EV treatment for neurodegenerative disorders were provided.

Multiple studies have reported that cancer cell-derived EVs target tumor tissues upon systemic delivery [80,81,91], exemplifying that EV source has an important role in targeting diseased tissues. For example, cancer cell-derived EV drug formulations had significantly delayed brain tumor growth in a GL26 brain tumor mouse model [79]. This was further shown with significant differences in the biodistribution of EVs derived from a muscle cell line (C2C12), a melanoma cell line (B16F10), and primary immature bone marrow-derived DCs that were detected post-systemic administration in mice [63]. Here, C2C12-derived EVs were found in greater concentrations within the liver compared to B16F10-EVs and DC-EVs, which had the lowest liver distribution. This was starkly contrasted with lung accumulation, in which B16F10-EVs and DC-EVs were found at higher levels than C2C12-EVs. Furthermore, B16F10-EVs were found more frequently in the GI tract relative to C2C12-EVs and DC-EVs. Intriguingly, DC-EVs had increased accumulation in the spleen relative to EVs from the other sources. A similar study demonstrated cell-type specificity for EVs released by murine colorectal cancer cells (C26) compared to murine melanoma cells (B16BL6) [81]. It was revealed that C26-EVs were taken up more efficiently by C26 cancer cells compared to allogeneic B16BL6-EVs in mice.

Nonetheless, special consideration should be given to employing cancer cell-derived EVs for drug delivery to tumors. For example, it has been demonstrated that cancer cell-derived EVs may also exert a broad array of detrimental effects, including angiogenesis, stromal remodeling [88], inactivation of the immune response to the tumor [89], inducing chemoresistance, and the formation of metastatic foci [82,90]. Therefore, “clean” EVs without content that could induce unwanted effects, especially in the case of cancer-derived EVs, may have considerable advantages. It is noteworthy that the removal of the “toxic” content from cancer cell-derived EVs should not introduce significant changes in the structure and content of their membranes. One of the solutions for the fabrication of “clean” EVs is bioinspired EV-mimetic nanovesicles that are produced by the breakdown of monocytes via serial extrusion through filters [92]. These cell-derived nanovesicles would be depleted from their internal content inherited from parental cancer cells.

In summary, the organotropism of EVs offers a promising path for targeted drug delivery, particularly in addressing neurodegenerative disorders. EVs tend to fuse preferentially with cells similar to their origin, likely influenced by surface integrins, enabling precise therapeutic delivery. Notably, EVs derived from neural stem cells have demonstrated significant potential in stroke models, improving neurological function and preserving tissue. These EVs have also been shown to reduce neuroinflammation, alleviate cognitive impairments in mouse models, and clear amyloid-β aggregates in AD models. Furthermore, while EVs from cancer cells have shown effectiveness in targeting tumor tissues, their potential harmful effects warrant careful consideration. Developing “clean” EVs or EV-mimetic nanovesicles could offer a safer alternative, harnessing the benefits of organotropism while minimizing associated risks.

### 3.2. Intrinsic Properties of Unmodified EVs

A growing body of evidence indicates that EVs contain biomolecules that are reflective of their cellular origin [63,93,94,95]. In this regard, the same repertoire of surface receptors and extracellular matrix-binding proteins may be acquired by EVs compared to their parental cells. In particular, it is well known that inflammation is involved across a spectrum of CNS disorders, including neurodegeneration (e.g., PD [96], AD [97]), stroke [98], brain cancer [99], infections [100], and brain trauma [101]. In this regard, immunocytes that include mononuclear phagocytes (e.g., dendritic cells, monocytes, and macrophages), neutrophils, and lymphocytes readily home to the sites of inflammation [102,103]. Therefore, EVs derived from inflammatory response cells could be used to target the inflammatory sites within the CNS often associated with neurodegenerative disorders and thus can be used as a CNS inflammatory-specific drug delivery systems. Notably, macrophage-derived EVs have been utilized in a number of studies for the treatment of CNS disorders, as macrophage-EVs have been shown to accumulate in the brain in regions of inflammation and neurodegeneration [44,45,47,48,49,65,104]. For instance, profound therapeutic effects in mouse models of PD and toxin-induced encephalitis were reported, followed by treatments with macrophage-derived EV-based formulations [48,49,77,105]. Monocyte-derived EVs have also been used for brain delivery of curcumin in an LPS-induced septic shock mouse model, where the enhanced anti-inflammatory activity of monocyte-EVs was recorded [103].

Regarding the mechanisms of targeting inflamed tissues, macrophage-derived EVs have a remarkable ability to communicate with recipient cells in inflamed mouse brain tissues via lymphocyte function-associated antigen 1/intercellular adhesion molecule-1 (LFA1/ICAM1) interactions [48,105]. Specifically, macrophage-derived EVs carry LFA-1 from their parental cell, and these LFA-1 associated EVs have been shown to interact with endothelial ICAM-1 receptor, which is overexpressed during times of inflammation to mediate the migration of immune cells and EVs across the BBB [105,106,107]. In one such study, macrophage-derived EVs were shown to deliver BDNF to the brain after IV injection [107]. Furthermore, a study compared the effect of EVs derived from macrophages (mEVs), neurons (nEVs), and astrocytes (aEVs) in a PD mouse model, in which the cell of origin greatly impacted targeting and effectiveness of treatment [65]. In this study, transgenic mice representing a PD model were treated with reporter mEVs, nEVs, or aEVs and then imaged over 480 h. In these mice, all treatments demonstrated the accumulation of EVs within the brain, with mEV accumulation the highest throughout all examined time ranges. They speculated this may have occurred due the high number of inflammatory targeting integrins (e.g., Integrin α4, Integrin αL, Integrin αM, and Integrin β) found on the mEVs compared to nEVs and aEVs.

Additionally, other molecules found on the surface of EVs have been shown to provide therapeutic advantages. This is seen with adhesive and immunomodulatory molecules expressed on the EV membrane [108,109,110,111]. For instance, mEVs were shown to have a significantly higher concentration of integrins and tetraspanin proteins involved in adhesion [108]. Furthermore, macrophage-derived EVs were reported to contain an abundance of immune molecules such as CD47 on their surface, which helps them escape immune attack [112] and enhances their immunomodulation properties [108]. Similar results have been shown with T cell-derived EVs, in which EVs released from T cells promote angiogenesis through a CD47-dependent manner on recipient endothelial cells [109]. It is noteworthy that a natural brain targeting ability of blood-borne EVs can be used for treatment of various neurodegenerative disorders, including PD [110]. In one study, blood-borne EVs loaded with dopamine successfully delivered the drug to the mouse brain upon IV administration, increasing dopamine levels 15-fold in the brain. It was hypothesized that blood-borne EVs could deliver drugs past the BBB based on the transferrin–transferrin receptor (TfR) interaction. Specifically, transferrin that is abundant in blood plasma [111] can bind tightly and exclusively to TfR, which is expressed at high levels on EVs derived from blood.

Overall, the impact of the biological origin of EVs is a crucial factor with respect to brain targeting ability. EVs released from different types of cells may differ in targeting and biological effects, which impact their therapeutic function and thus parental cells should be considered based on the desired therapy [63,113]. The choice of the cell source for EV derivation is crucial, particularly in vivo when the EV source can determine organ targeting. Thus, researchers must consider both the donor cell phenotype as well as the characteristics of the EVs when choosing an EV delivery system for enhanced therapeutic efficacy.

## 4. Targeting CNS with Brain-Specific Ligands

### 4.1. Conjugation of Vector Moieties on the Surface of EVs

Much effort has been dedicated to the development of modified EVs by adding specific ligands or antibodies to their surface that bind to receptors on recipient cells to further enhance their ability to target the CNS. The most exploited vector moiety is the neuron-specific rabies viral glycoprotein (RVG) peptide that can specifically bind to the acetylcholine receptor expressed by neuronal cells and cells of the BBB [114,115,116,117,118,119,120,121,122,123,124]. In one of the first reports, investigators utilized EVs released by transfected parent dendritic cells to express lysosomal-associated membrane protein 2b (Lamp2b) fused to RVG peptide for AD therapy [114]. Purified RVG-Lamp2b-EVs were loaded with exogenous short interfering (si)RNA to GAPDH by electroporation. Intra-tail injection of drug-loaded RVG-Lamp2b-EVs resulted in significant knockdown of GAPDH mRNA in several regions of the brain and protein knockdown of BACE1, a therapeutic target in AD. Recently, a similar approach has been applied to EVs released by human-induced pluripotent stem cells (iPSCs) that were genetically engineered to overexpress RVG-Lamp2b-HA using CRISPR/Cas9-assisted homologous recombination [115]. Tang et al. utilized a mouse model to demonstrate the enhanced targeting abilities of systemically administered RVG-Lamp2b-modified EVs, which had superior brain targeting compared to control EVs, and suggested this targeting was due to the RVG modification of EV membrane components (e.g., phospholipids). In another study, MSC-derived RVG-EVs were tested in an AD, transgenic APP/PS1 mouse model [119]. Here, EVs were conjugated with RVG through a DOPE-NHS linker; these RVG-EVs were then administered via IV injection and were shown to accumulate in the hippocampus and cortex of the treated mice. Furthermore, RVG-EVs were shown to significantly decrease proinflammatory cytokines (TNF-α, IL-β, and IL-6) while significantly increasing the expression of anti-inflammatory cytokines (IL-10, IL-4, and IL-13). Importantly, RVG-EV intervention resulted in a significant improvement in learning and memory capabilities with sharp decreases in plaque deposition and Aβ levels, and a reduction in astrocyte activation in transgenic APP/PS1 mice.

A number of investigations have utilized RVG-EVs for the treatment of PD. RVG-vectorized EVs were used for the brain delivery of alpha-synuclein (α-Syn) siRNA to lower the total and aggregated levels of α-Syn in a mouse model of PD [116]. Systemically injected RVG-EVs loaded with α-Syn-siRNA decreased α-Syn mRNA and reduced intraneuronal protein aggregates, even within dopaminergic neurons of the substantia nigra throughout the brain of S129D α-Syn transgenic mice.

One of the drawbacks of siRNA treatment is its short-term efficacy. Therefore, α-Syn -shRNA mini circles (shRNA-MCs) to were suggested to treat the α-Syn preformed fibril-induced model of parkinsonism for prolonged effectiveness [121]. Systemic administration of RVG-EVs loaded with shRNA-MCs reduced α-Syn aggregation, decreased the loss of dopaminergic neurons, and led to improvement of clinical symptoms. Furthermore, DNA aptamers that specifically recognize α-Syn were delivered by means of RVG-EVs to the mouse brain [120]. Thus, IP administration of the aptamer-loaded RVG-EVs significantly lowered α-Syn preformed fibril (PFF)-induced pathological aggregates and reversed synaptic protein loss as well as neuronal death. Finally, a complex formulation of DC-derived RVG-EVs loaded with two therapeutic modalities, curcumin/phenylboronic acid-poly(2-(dimethylamino)ethyl acrylate) nanoparticle and siRNA targeting *SNCA*, an α-Syn protein coding gene, was employed as a nano-scavenger for the clearance of α-Syn aggregates and for reducing its cytotoxicity [122]. The effective delivery of siRNA and chemical drugs by RVG-EVs reduced the α-Syn aggregates in diseased dopaminergic neurons and induced immune tolerance that resulted in the reconstruction of immune homeostasis in the PD mouse model.

Delivery of siRNA via RVG-EVs has also been applied to treat other neurodegenerative disorders. For example, brain-targeted RVG-EVs were employed to transport miR-193b-3p to the brain of mice with subarachnoid hemorrhage (SAH) which typically results from a ruptured aneurysm [123]. Bone marrow mesenchymal stem cells (BMSCs) were transfected to express Lamp2b fused with the targeting peptide RVG and loaded with miR-193b-3p by electroporation. SAH mice that received treatment with miR-193b-3p-loaded RVG-Lamp2b-EVs were shown to have attenuated expression of histone deacetylase 3 (HDAC3), which in turn upregulated acetylated NF-κB p65 and decreased proinflammatory cytokines leading to improved neurological behavior and the reduction in brain edema and BBB injury. Likewise, RVG-EVs have also been employed for siRNA delivery of microRNA-124 (miR-124) to the infarct site in a mouse model of cortical ischemia [118]. Yang et al. demonstrated that miR-124-loaded RVG-EVs protected mice against ischemic injury through the promotion of neuronal identity in neural progenitors and the promotion of cortical neurogenesis. RVG-EVs have also been used for brain delivery of opioid receptor mu (MOR) siRNA to treat morphine addiction [117]. It was demonstrated that the delivery of MOR siRNA by RVG-EVs inhibited morphine relapse in mice by downregulating the expression levels of MOR in the brain upon a single IV injection. Next, engineered EVs targeted to the brain with RVG fused to Lamp2b were utilized for the fabrication of EV-coated gold nanoparticles (AuNPs) for theragnostic applications [124]. Bioluminescence imaging revealed that RVG-Lamp2b-EV-coated AuNPs accumulated in the mouse brain after IV injection.

In addition to the RVG peptide, EVs have also been targeted to the ischemic brain with the c(RGDyK) peptide conjugated to the surface of the EVs by linkage to azide-containing molecules via copper-free click chemistry [125]. Curcumin-loaded RGD-EVs were IV-injected into ischemic mice; these EVs were shown to target the ischemic lesion within the brain resulting in a reduction in inflammation and cellular apoptosis.

EVs can also target specific neuronal populations due to the presence of unique surface markers. These molecules allow them to bind to receptors on distinct types of neurons, effectively delivering their associated cargo (e.g., proteins, RNA, lipids) to those specific neuronal populations within the brain, making them a potential therapeutic tool for neurodegenerative diseases. A novel therapeutic system of NSC-derived EVs were modified with increased expression of the ligand PDGF-A (EVPs) for the therapy of CNS demyelinating diseases, as well as for neuroinflammation [126]. Parental NSCs were modified to express the ligand PDGF-A which targets the myelinating cells, or oligodendrocytes (OLGs), in the CNS. These EVPs were further modified through the addition of triiodothyronine (T3), a thyroid hormone that plays a role in the development of oligodendrocytes. In this study, Xiao et al. [126] demonstrated the increased efficiency of these T3-modified EVPs in the targeting of OLGs in an experimental autoimmune encephalomyelitis model upon IV injection of PDGF-A-EVs. Furthermore, T3-modified EVs were shown to alleviate disease development, promote the survival of OLGs, promote myelin regeneration, and inhibit myelin damage.

Along with EVs targeted to the brain, these natural carriers have also been vectorized to glioma cells and the tumor vascular endothelium [127,128,129,130]. For example, EVs collected from the macrophage cell line Raw264.7 media have been conjugated with a neuropilin-1-targeted peptide (RGERPPR, RGE) by click chemistry [128]. Drug-loaded RGE-EVs with imaging and therapeutic functions were used for superparamagnetic iron oxide nanoparticle (SPION)-mediated magnetic flow hyperthermia (MFH) and curcumin-mediated therapy. SPIONs and curcumin were loaded into RGE-EVs by electroporation and RGE peptide was conjugated to cargo-loaded EVs by click chemistry with a cycloaddition reaction of sulfonyl azide. IV-injected RGE-EVs concentrated in the tumor region more effectively, indicating their ability to cross the BBB, target the glioma cells, and improve their residence time in the tumor area when compared with non-targeted EVs. RGE-EV-SPION/Cur also demonstrated a synergistic effect of the SPIONs and curcumin which had significant inhibitory effects on the growth of glioma cells and orthotopic xenografts. 

Additionally, several studies have utilized an angiopep-2 (Ang-2) peptide for enhancing drug delivery to GBM. For example, multifunctional exosomes-mimetics (EM) were decorated with Ang-2 for GBM therapy [127]. Antineoplastic agent docetaxel (DTX) loaded into these bioinspired nanocarriers showed significant inhibition on orthotopic GBM growth with reduced side effects. Furthermore, dual-receptor-specific EVs loaded with temozolomide (TMZ) and O^6^-benzylguanine (BG) were reported for eradicating TMZ-resistant GBM [129]. Target ligands Ang-2 and CD133 RNA aptamers were conjugated on the surface of EVs using an amphiphilic molecule bridge that was expressed through the induction of donor cells. To evaluate the anti-glioma efficacy, BALB/c nude mice bearing GMB U87MG were IV-injected with Ang-2-CD133 Apt-EV-TMZ and Ang-2-CD133 Apt-EV-BG. Dual targeting significantly enhanced BBB penetration and provided superior tumor accumulation of Ang-2-CD133 Apt-EVs. The combination of TMZ and BG in targeted EVs dramatically increased their efficacy compared with that of free drugs. In another study, similar vector molecules, Ang-2 and CD133-targeted peptides, were introduced to GBM cell-derived EVs to improve their capacity for penetrating the BBB and targeting tumor cells [130]. Ang-2-CD133-EVs were loaded with the chemotherapeutic agents TMZ and DOX. It was reported that drug-loaded EVs crossed the BBB, targeted GBM cells, penetrated deep into the tumor parenchyma, and released their therapeutic cargos. This synergistic TMZ/DOX delivery suppressed the tumor growth and prolonged survival time in the orthotopic syngeneic mouse model of GBM.

Besides targeting GBM, Ang-2-modified EVs have also been used for treatment of CNS tuberculosis (CNS-TB) [131]. Ang-2-EVs were employed as nanocarriers for antimicrobial agent rifampin (RIF). EVs were isolated from BMSCs, loaded with RIF by electroporation, and modified with Ang-2 by chemical reaction. Compared with untargeted EVs, IV-injected Ang-2-EVs were preferentially accumulated in the brain tissues and increased the concentration of RIF in the brain.

### 4.2. Prolonged Circulation of EVs in the Blood Stream

With respect to enhancing targeted therapeutic delivery, efficacy, and safety, EVs offer unique advantages compared to other types of carriers, leading to a longer circulatory half-life and decreased clearance by RES. It has been demonstrated that enhanced retention of EVs, compared to liposomes or other synthetic nanocarriers, in the circulation of mice is likely due to the specific expression of CD47 on their surface that provides the protection of EVs from phagocytosis by monocytes and macrophages [112]. To further improve the circulation lifetimes and targeting ability, EVs were modified with surface polyethylene glycol (PEG) [132]. The biodistribution of copper-64 (^64^Cu)-radiolabeled-PEG-modified EVs was examined in vivo using positron emission tomography (PET). It was clearly demonstrated that PEGylation endowed EVs with a superior pharmacokinetic profile and reduced premature hepatic sequestration and clearance of EVs in mice. However, one should take into consideration that PEGylation of EVs might diminish interactions between specific binding molecules on their surface and receptors on target cells.

Overall, these findings, summarized in Table 2, promise enhanced therapeutic delivery, especially for targeted EV-based formulations. However, despite these successes, achieving efficient EV targeting via surface modification is not a trivial matter and requires careful consideration during development followed by validation studies to ensure success.

## 5. Using External Stimuli for Brain Targeting

The approaches that utilize an external force for targeting EV-based drug formulations to the brain are not fully developed. In fact, we were able to find only one report regarding the use of a magnetic field to guide drug-loaded EVs to the brain [133]. To accomplish brain accumulation, EVs were manufactured using parental MSCs harboring iron oxide nanoparticles (IONPs). Interestingly, in addition to magnetic properties, IONP-loaded EVs contained significant amounts of therapeutic growth factors due to the stimulation of their expression in parental MSCs. Specifically, a quantitative real-time polymerase chain reaction (qRT-PCR) assay demonstrated that treatment with IONPs increased the mRNA expression levels of therapeutic molecules related to angiogenesis (e.g., angiopoietin 1 (Ang-1), fibroblast growth factor 2 (FGF2), hepatocyte growth factor (HGF), vascular endothelial growth factor (VEGF), platelet-derived growth factor (PDGF), and transforming growth factors ((TGF)-β1 and TGF-β3), as well as anti-apoptosis (e.g., FGF2, HGF, VEGF, PDGF, TGF-β1, TGF-β3), neurotrophic properties (e.g., nerve growth factor (NGF), brain-derived neurotrophic factor (BDNF), glial-cell-derived neurotrophic factor (GDNF), neurotrophin-3 (NT3), NT4), and anti-inflammation (e.g., TGF-β1, TGF-β3) in MSC/IONP. After systemic injection of IONP-EVs into transient middle-cerebral-artery-occlusion (MCAO)-induced rats, the magnetic navigation significantly increased their accumulation in the ischemic lesion. Additionally, IONP-EV treatment improved the anti-inflammatory response, angiogenesis, and anti-apoptosis. This resulted in a reduced infarction volume and enhanced motor function in MCAO-induced rats.

External magnetic field could also target EV-based formulations to tumors. Thus, in vivo studies of murine melanoma subcutaneous cancer models have demonstrated that the delivery of TNF-α-loaded EVs improved cancer targeting under an external magnetic field and diminished tumor growth with mitigating toxicity [134]. Furthermore, using ferromagnetic nanotubes for magnetic-field-controlled EVs delivery upon application of an external magnetic field was investigated in a murine model of Duchenne muscular dystrophy [135] and infarcted heart tissues [136]. In vivo models of myocardial infarction under magnetic guidance led to the accumulation of CD63-expressing EVs in infarcted tissue and decreases in infarct size and improved left-ventricle ejection fraction. A similar strategy may be applicable to control the accumulation of EVs in the brain.

## 6. Challenges Surrounding EV-Based Therapeutics

### 6.1. Scalability and Standardization of EV Production

An outstanding challenge for the future development of EV-based therapeutics is having robust and reproducible manufacturing platforms to ensure consistency in EV production. The production of EVs in therapeutic quantities requires large-scale and high-capacity protocols that do not compromise the overall quality and function of the final product [137,138]. Although a majority of EV-based research is limited to small-scale cultures produced from flasks, a number of strategies to upscale EV production, such as 3D culture, physical stimulation, chemical stimulation, and the genetic modification of producing cells have been examined [139]. In particular, 3D cell culture using bioreactor systems is a revolutionary technology that offers significant benefits for large-scale EV production. Bioreactor-based systems are advantageous due to their scalability, increased capacity, and controlled culture environment which more closely resemble physiological conditions [140]. Along these lines, the use of hollow fiber bioreactor systems for EV production has recently been explored [140,141,142]. For example, Cao et al. found that MSC-EVs produced from a three-dimensional hollow fiber bioreactor were approximately 15.5 more concentrated than EVs produced in 2D flasks. Additionally, the EVs produced from the bioreactor displayed enhanced reparative and anti-inflammatory properties in a mouse model of cisplatin-induced acute kidney injury [143]. In another study, MSC-EVs produced in a hollow fiber bioreactor had increased yield and increased functional effects on chondrocytes in vitro, as well as improved cartilage repair in vivo [144]. Others have also employed similar fiber-based systems to improve the yield and concentration of EVs from different cell types [145,146,147,148]. Scalable technology also exists for EV isolation, namely, tangential flow filtration (TFF). During the process of TFF, biofluids flow tangentially across the surface of a porous filter membrane, while a pressure gradient is applied to the membrane to drive the separation of EVs. Coupled with the upstream culturing techniques to increase EV yield described above, TFF can further boost the yield, consistency, and purity of isolated EVs. To this end, we and others have reliably used TFF to produce functional EVs that have demonstrated widespread reparative properties in various cell types, including those of the CNS [149,150,151,152,153]. Importantly, TFF technology is compatible with current Good Manufacturing Practices (cGMPs), which are a set of guidelines that are maintained and enforced by the Food and Drug Administration (FDA) and apply to all aspects of therapeutic production. Collectively, these studies provide a strong foundation for the continued optimization and standardization of large-scale production strategies for EV-based therapeutics.

### 6.2. Cargo Loading Efficiency

Another well-known challenge surrounding EV-based therapeutics is the efficient loading of cargo without inducing significant changes in the structure and content of the EV membrane. Due to the dense membrane of EVs, cargo loading of EVs has poised more challenges than the loading of their close relatives, synthetic vesicles such as liposomes and polymer-based nanoparticles [154]. Indeed, EVs have well-organized and tight lipid-bilayer membranes that greatly restrict drug penetration, so unlike synthetic vesicles, EVs therapeutics should be loaded into EVs after their formation. In addition, substantial precautions should be considered during drug loading to preserve the integrity of the EV membrane to protect crucial proteins on the EV surface. These factors impose substantial restrictions on EV drug incorporation methods and, as a result, impede the overall loading efficacy of EV-based drug formulations. Another point worth considering is that disruption of EV membrane integrity during drug loading may alter the “immune-privileged” status of the EVs, thereby making them visible for the mononuclear phagocyte system (MPS). Herein lies the challenge in which loading EVs with cargo may disrupt the natural biological activity that is associated with EV delivery due to changing the composition of the membrane and potentially diminishing the intended therapeutic effect.

Overall, two distinct approaches can be utilized for the loading of EV carriers with therapeutic cargo: (*i*) exogenous loading of EVs (i.e., loading naïve EVs isolated from parental cells ex vitro) and (*ii*) endogenous loading of EVs (i.e., parental cells are loaded with either the drug or transfected with DNA encoding the therapeutic molecule of interest, and the subsequent EVs released contain the drug or active molecules). Each approach has its own advantages and limitations, which may also be influenced by the properties of the therapeutic cargo, the site of the diseased tissue, and the specific conditions required to achieve efficient loading. While an in-depth review of these strategies is outside the scope of the current review, several other excellent reviews have explored these topics in depth [155,156,157,158].

## 7. Conclusions and Future Perspective

Overall, EVs show remarkable promise as therapeutic drug delivery systems for neurodegenerative disorders due to their high bioavailability as well as high biocompatibility and low immunogenicity. Furthermore, EVs have shown a strong ability to target organs (such as the brain) and are able to travel across physiological barriers (i.e., BBB) which demonstrates that EVs may provide a powerful and novel delivery platform for the therapy of CNS diseases and neurodegenerative disorders. There are still technological, functional, and safety challenges that must be addressed before the use of EVs as therapeutic delivery systems, such as large-scale production and cargo loading. Nevertheless, this review highlights the major findings that provide optimism for the development of EV-based formulations as a vehicle for targeted and drug delivery to the CNS.

## Figures and Tables

**Table 1:** Organotropism of EVs. This table summarizes the key points about EV organotropism, such as the effects of primary stem cell and cancer cell EVs on targeting and therapeutic effects. Here, primary cell-derived EVs such as hNSC-EVs were shown to reduce neuroinflammation in a variety of models, while cancer cell-derived EVs were found to delay tumor growth; there were additional detrimental effects observed.

Donor Cell	Disease Model	Major Findings	Study
Human Neural Stem Cells (hNSCs)	Stroke (pig, murine, and rat models)	Enhanced neurological recovery. Of note, IFN-γ-hNSC-EVs showed better outcomes.	[83,84,85]
	Chronic neural inflammation (LPS-induced in mice)	hNSC-EVs were shown reduce microglial activation and inflammation with marked reduction in proinflammatory NLRP3 pathway.	[86]
Mesenchymal Stem Cells	Stroke (murine model)	MSC-EVs improved neurological recovery. However, MSC-EVs were less effective than NSC-EVs.	[84]
Neuronal Cells vs. Glial cells	AD (Aβ aggregation in AAPP mice)	EVs from neuronal cells (not glial cells) were shown to capture and clear Aβ as well as reduce amyloid deposits.	[87]
Cancer Cells	Brain tumor (GL26 mouse model)	Delayed brain tumor growth; cell-type specificity observed in EV biodistribution.	[79]
	EV biodistribution in various tumor murine models	EVs from different cancer cell lines were found to have distinct tropism and may have detrimental effects (e.g., promoting angiogenesis, inducing chemoresistance).	[63,81,82,88,89,90]

**Table 2:** EVs modifications with ligands for CNS targeting. This table summarizes key findings from in vivo studies utilizing ligand modifications of EVs (i.e., rabies viral glycoprotein (RVG) and neuropilin-1 targeted peptide (RGE)) for CNS targeting via systemic administration in mice models. Ligands such as RVG and RGE peptides have demonstrated enhanced ability to target the CNS across multiple studies when incorporated into EVs. Studies have shown a preferential binding of RVG to neuronal nicotinic acetylcholine receptors, which are abundantly expressed on neurons. Similarly, RGE has been shown to target tumor environments within the CNS (e.g., glioma cells).

Disorder	Donor Cell	Ligand	Loading Mechanism	Other EV Modifications	Major Findings	Study
Targeted Delivery	HEK293T	RVG-Lamp2b	Transfection	Gold nanoparticles were mechanically loaded	RVG-EVs effectively carried AuNPs to the brain.	[124]
	human iPSCs	RVG-Lamp2b-HA	CRISPR/Cas9-assisted homologous recombination	Labeled with NIR dye	RVG-EVs have improved targeting to the brain compared to EV controls.	[115]
AD	Murine dendritic cells	Lamp2b-RVG	Transfection	*GAPDH* siRNA loaded via electroporation	Significant knockdown of GAPDH mRNA throughout the brain. Knockdown of protein BACE1.	[114]
	MSC	DOPE-RVG	DOPE-NHS linker	Labeled with lipophilic dye DiI	Significant decrease in proinflammatory cytokines and increase in anti-inflammatory cytokines. Improved learning and memory capabilities. Decreased plaque depositions, Aβ levels, and astrocyte activation.	[119]
PD	Murine dendritic cells	RVG-Lamp2b	Transfection	α-Syn siRNA loaded via electroporation	Delivery of EV-associated siRNA to the brain. Mice had decrease in α-Syn mRNA and protein.	[116]
	Murine dendritic cells	RVG-Lamp2b	Transfection	shRNA-mini circles (MC) loaded via electroporation	Decrease in α-Syn aggregation, reduction in loss of dopaminergic neurons, improved clinical symptoms.	[121]
	HEK293T	RVG-Lamp2b	Transfection	α-Syn aptamer loaded via transfection	Aptamer-loaded EVs were delivered into neurons, reduced α-Syn PFF, and reduced the loss of dopaminergic neurons.	[120]
	Murine dendritic cells	RVG	Ultrasonic assembly	C/ANP/S core loaded via ultrasonic assembly	RVG-EVs with C/ANP/S cores were shown to decrease α-Syn and improved motor behavior in mice.	[122]
SAH	Murine BM-MSCs	RVG-Lamp2b	Transfection	FAM-labeled miR-193b-3p or scrambled miRNA were loaded via electroporation	RVG-EVs with miR-193b-3p were more effective at delivery of miR-19b-3p to the sight of injury compared to miR-19b-3p alone, and reduced behavioral impairment, brain edema, BBB injury, and neurodegeneration.	[123]
Cortical ischemia	Murine BM-MSC	RVG-Lamp2b	Transfection	Fluorescently labeled; miR-124 or scrambled miRNA were loaded via electroporation	RVG-EVs loaded with miR-124 promoted neuron differentiation and protected ischemic injury.	[118]
Morphine addiction	293T	RVG-Lamp2b	Transfection	MOR siRNA transfection fluorescence-labeled siRNA	RVG-EVs efficiently transfer siRNA to CNS and downregulate MOR expression inhibiting morphine relapse.	[117]
Cerebral Ischemia	Murine BM-MSC	c(RGDyK) peptide	Bio-orthogonal chemistry	Curcumin and triiodothyronine (T3) loaded	Reduction in inflammatory response and apoptosis near the lesion.	[125]
Autoimmune Encephalomyelitis	Murine BM-NSC	PDGF-A	Transfection	Via sonication	Slowed down disease development by reducing myelin damage and promoting oligodendrocyte survival and myelin regeneration.	[126]
Glioma	U87-MG	Angiopep-2	Conjugation with DSPE-PEG2000 as a linker	Plasma membrane of EVs were isolated and used to synthesize liposomes via extrusion and loaded with docetaxel	Docetaxel loaded “exo-mimics” showed increased ability to deliver DTX to the tumor area and reduced GBM growth.	[127]
	Raw264.7 cells	Neuropilin-1-targeted peptide (RGE)	Click chemistry	SPION/Cur were loaded via electroporation	Synergistic anti-tumor effect with SPIONs and Cur, with increased delivery and decreased therapeutic side effects when delivered via RGE-labeled EVs.	[128]
	THP-1	Angiopep-2 and CD133 RNA	Amphiphilic molecule bridge	Temozolomide (TMZ) and O^6^-benzylguanine (BG) were loaded via sonication	Extended life in mice with less side effects than therapeutics alone.	[129]
	U251 GBM	Angiopep-2 and CD133 RNA	Click chemistry	Temozolomide (TMZ) and O^6^-benzylguanine (BG) were loaded via sonication	Were able to penetrate tumor environment and suppress tumor growth, increasing survival time in mice.	[130]
CNS-TB	BMSCs	Angiopep-2	Click chemistry	Rifampin (RIF) loaded via electroporation	Higher targeting capacity compared to unmodified EVs; furthermore, modified EVs did not change the MIC or MBC (although this was determined in vitro).	[131]

## Data Availability

All data is derived from references cited and found on PubMed.

## Abbreviations

The following abbreviations are used in this manuscript:

AAV	Adeno-associated Virus
AD	Alzheimer’s Disease
Ang-1	Angiopoietin 1
BBB	Blood–Brain Barrier
BCSFB	Blood–Cerebrospinal Fluid Barrier
BDNF	Brain-derived Neurotrophic Factor
BME	Brain Microvessel Endothelium
BMSCs	Bone Marrow Mesenchymal Stem Cells
cGMPs	current Good Manufacturing Practices
CNS	Central Nervous System
CSF	Cerebrospinal Fluid
CuSNPs	Copper Sulfide Nanoparticles
DOX	Doxorubicin
FDA	Food and Drug Administration (FDA)
FGF2	Fibroblast Growth Factor 2
GBM	Glioblastoma Multiforme
GDNF	Glial-cell-derived Neurotrophic Factor
GI	Gastrointestinal Tract
HD	Huntington’s Disease
HGF	Hepatocyte Growth Factor
hNSCs	Human Neural Stem Cells
IC	Intracranial
IN	Intranasal
LINCL	Late-infantile Neuronal Ceroid Lipofuscinosis
LPS	Lipopolysaccharide
MCAO	Middle-Cerebral-Artery-Occlusion
MPS	Mononuclear Phagocyte System
MSCs	Mesenchymal Stromal Cells
NHP	Non-human Primates
NT3	Neurotrophin-3
OPC	Oligodendrocyte Precursor Cell
PBS	Phosphate-buffered Saline
PD	Parkinson’s Disease
PDGF	Platelet-derived Growth Factor
PEG	Polyethylene Glycol
qRT-PCR	Real-time Polymerase Chain Reaction
RES	Reticuloendothelial system
RIF	Rifampin
RVG	Rabies Viral Glycoprotein
SAH	Subarachnoid Hemorrhage
SC	Subcutaneous
SCI	Spinal Cord Injury
SPION	Superparamagnetic Iron Oxide Nanoparticle
TBI	Traumatic Brain Injury
TFF	Tangential Flow Filtration
TfR	Transferrin Receptor
TGF	Transforming Growth Factor
TME	Tumor-microenvironment
TMZ	Temozolomide
TPP1	Tripeptidyl Peptidase-1
Tregs	Regulatory T cells
VEGF	Vascular Endothelial Growth Factor
